# Synthesis and structure of metal-TCPE (metal = Th, Ce) metal-organic frameworks based on 1,2,4,5-tetrakis(4-carboxyphenyl) ethylene

**DOI:** 10.1098/rsos.220525

**Published:** 2022-08-31

**Authors:** Lin Li, Ting Yu, Zhenghua Qian, Xiaoling Wu, Hui He, Guoan Ye, Yanbo Qiao

**Affiliations:** ^1^ Shanghai Institute of Applied Physics, Chinese Academy of Sciences, Shanghai 201800, People's Republic of China; ^2^ University of Chinese Academy of Sciences, Beijing 100049, People's Republic of China; ^3^ Department of Radiochemistry, China Institute of Atomic Energy, Beijing 102413, People's Republic of China

**Keywords:** Th(IV), Ce(IV), MOFs, tetradentate ligand, luminescence

## Abstract

Two new metal-organic frameworks (MOFs) (Th/ Ce -TCPE) based on 1,2,4,5-tetrakis(4-carboxyphenyl)ethylene were obtained using a straightforward reaction under moderate conditions. Th and Ce formed the central units of this MOF in the mononuclear and in the unusual trinuclear cluster configurations, respectively. The resulting MOFs were analysed by fluorescence spectroscopy to understand their luminescence. The obtained data revealed that benzene's electron cloud density and torsion angle on the ligand were affected by the acetic acid molecule and Th(IV), which caused Th-TCPE to irradiate stronger blue emission, but Ce-TCPE showed no fluorescence due to the self-quenching. Such a unique luminescence property could be used for fluorescence or radiopharmaceutical sensing.

## 1. Introduction

Actinides are by-products of spent fuel reprocessing, recycling and separation. Due to the unfilled 5f and 6d electron shells, the physical and chemical properties of actinides are unique [1–3], which attracts scientists worldwide to study compounds of tetravalent actinides including thorium, uranium and plutonium [4,5]. Yet, due to the complexity of their chemistry, the structure, chemical bonding nature and reaction mechanisms of these compounds are still not fully understood [6,7].

Materials based on metal-organic frameworks (MOFs), which are organic–inorganic nanoporous hybrids, have emerged in recent years. They are composed of coordinated metal ions and organic ligands [8,9]. With the synthesis and application of a large number of uranium-based MOFs, thorium-based MOFs has also received extensive attention. For example, O'Hare *et al*. [10–12] synthesized one-dimensional hexagonal nanotube-like thorium-based MOFs (TOF-1) with 1,3,5-benzotriformic acid as organic ligand. This group also synthesized TOF-2 and TOF-3 with different size cages. Thierry *et al*. [13] used a bidentate ligand to synthesize Th-UiO-66, which was later functionalized by Li *et al*. [14,15] to synthesize a Th-UiO-66-X series of compounds. Islamoglu *et al*. [16] combined Th with a tridentate ligand and obtained Th-MOF-808, while Li *et al*. [17] combined Th with a tetradentate ligand to obtain Th-PCN-222. Th-based MOFs also exhibit the capability of fluorescent properties. In our previous work, we used H4TCPB to synthesize several fluorescing Th-based MOFs [18]. Fluorescing MOFs based on other tetravalent actinides were also obtained by other groups [19,20].

This paper reports research data obtained as a continuation of those efforts. We used Th(IV) and Ce(IV) nitrates (Ce as a representative tetravalent actinide) and 1,2,4,5-tetrakis(4-carboxyphenyl)ethylene (H4TCPE) ligand to synthesize Th(IV)-based Th-TCPE (compound **1**) and Ce(III)-based Ce-TCPE (compound **2**) MOFs [21]. Ce(IV) is reduced to Ce(III) in this process, and it is speculated that other tetravalent actinides may also be the case such as Pu(IV). Both compounds were thoroughly characterized. Compound **1** enhanced the luminescence intensity of the ligand, while compound **2** did not exhibit fluorescing properties. Some explanations are given for the mechanism of this phenomenon.

## 2. Experimental section

Caution! ^232^Th used in this study is radioactive and toxic. The standard procedure of radioactive material handling should be strictly obeyed during the preparation of the materials below.

### 2.1. Material and methods

In this work, we used Th(NO_3_)_4_·5H_2_O (99.5%) purchased from Shanghai Aladdin Biochemical Technology Co., Ce(NO_3_)_4_ (99.9%) supplied by Shanghai Macklin Biochemical Co., H4TCPE (98%) from the Jilin Chinese Academy of Sciences-Yanshen Technology Co., and N, N′-Dimethylformamide (DMF, 99.5%) acquired from Shanghai Meirel Chemical Technology Co. We also used glacial acetic (99.5%) and formic (88%) acids as well as ethanol (EtOH, 99.7%) supplied by Sinopharm Chemical Reagent Co. and normal hexane (95%) purchased from Shanghai Honeywell Trading Co.

### 2.2. Synthesis and structural determination

Compound **1** (Th-TCPE): 11.40 mg of Th(NO_3_)_4_·5H_2_O, 10.17 mg of H4TCPE and 0.40 ml glacial acetic acid were mixed with 1.5 ml of DMF in a 7 ml vial, which was then sealed and heated to 80°C for 48 h. The absinthe-green crystalline powder was filtered to separate it from the mother solution, washed three times with 30 ml of DMF, three times with 30 ml of EtOH and dried at room temperature. The amounts of these reagents were reduced 10 times to synthesize single crystals using the same procedure. The yield was 65.2% recalculated relative to the initial H4TCPE amount. The elemental analysis (EA) confirmed that the formula of the synthesized compounds could be expressed as Th_4_(TCPE)_2_(C_2_H_3_O_2_)_8_(C_3_H_7_NO)_3_(H_2_O)_20_. The molecular weight is 2409.36. The theoretical C, H and N weight contents in this compound (equal to 33.89%, 3.73% and 1.53%, respectively) corresponded well to those obtained from the experimental analyses (equal to 34.12%, 3.45% and 1.66%, respectively).

Compound **2** (Ce-TCPE): 10.25 mg of Ce(NO_3_)_4_, 10.17 mg of H4TCPE, 0.05 ml of glacial acetic and 1.5 ml of DMF were sealed in a 7 ml contained and heated at 100°C for 12 h. The resulting powder was filtered, washed three times with 30 ml of DMF, then three times with 30 ml of EtOH and dried at room temperature. The amounts of these reagents were reduced 10 times to synthesize single crystals using the same procedure. The final product yield was 62.2%, calculated relative to the initial H4TCPE amount. The theoretical C, H and N weight contents calculated for the resulting Ce(IV)Ce(III)_2_(μ-OH)_2_(TCPE)_2_(C_3_H_7_NO)_4_(H_2_O)_5_ (the molecular weight is 1461.22) compound should be equal to 46.86%, 3.80% and 3.04%, respectively. Corresponding C, H and N contents determined analytically were equal to 46.69%, 3.67% and 2.78%, respectively.

### 2.3. Characterization

*Single-crystal X-ray diffraction (SCXRD)* was performed using a Bruker APEX-II CCD diffractometer coupled with a Turbo X-ray source relying on Ga K*α* radiation at *λ* = 1.34139 Å. The instrument contained a direct-drive rotating anode and a CMOS detector. The crystal temperature was maintained at 173.0 K and 189.99 K during data acquisition. We used Olex2 [22] to solve the structure by an intrinsic phasing method. The data were then refined by ShelXL [23] software using the least square minimization approach. The scattering contribution due to the highly disordered ligand molecules was adjusted by the SQUEEZE simulation package of the PLATON program. All crystallographic data and some bond lengths are given in the electronic supplementary material, tables S1–S3.

*Powder X-ray diffraction (PXRD)* spectra were collected in the 5–50° range with a 0.02° step using a Bruker D8 Advance instrument equipped with Cu K*α* radiation with *λ* = 1.54178 Å as an X-ray source. First, we calculated (using SHELXTL-XPOW software) theoretical PXRD patterns using the data from the CIF files. Then, the experimental PXRD data were compared to these patterns to confirm the crystallinity and purity of the obtained compounds (see electronic supplementary material, figure S1).

*Infrared spectroscopy (IR)* was performed in the 2500–400 cm^−1^ range using a Thermo Scientific Nicolet iS20 instrument coupled with a diamond attenuated total reflectance set-up (see electronic supplementary material, figure S2).

*Scanning electron microscopy (SEM)* was performed by a Zeiss Merlin Compact LEO 1530 VP instrument. The compound **1** and **2** morphologies were oval and approximately 20 µm in size and columnar and 20–50 µm in size, respectively (see electronic supplementary material, figure S3).

EA was performed by the CHN model of the Elementar Vario EL III instrument.

*Thermogravimetric analysis (TGA)* was conducted using a Mettler-Toledo instrument under static N_2_. The samples were heated up to 700°C with a 10°/min rate (figure 4).

*Surface area and porosity measurements* were performed by recording the N_2_ and Ar adsorption/desorption isotherms at 77 K and 87 K by a Micromeritics ASAP 2020 instrument. Before the tests, compounds were soaked in n-hexane for 6 h to exchange DMF and then placed into sealed quartz crucibles, followed by their evacuation to 10^−2^ kPa and consequent heating at 100°C for 6 h (see electronic supplementary material, figure S4).

*Fluorescence spectroscopy* was recorded by an Edinburgh FLS spectrophotometer. Quantum yield (QY) was obtained using the Hamamatsu QY-plus set-up (figure 6).

X-ray photoelectron spectroscopy (XPS) was recorded by the full spectrum data measurement and analysis of the valence of Ce in compound **2** by a Thermo Fisher Nexsa (see electronic supplementary material, figure S5).

*In situ XRD* spectra were collected from 25°C to 700°C using a PANalytical Sharp Shadow instrument in the 5–50° range with a 0.02° step (see electronic supplementary material, figure S6).

## 3. Results and discussion

### 3.1. Crystal structures

#### 3.1.1. Compound **1**

**Figure 1.**
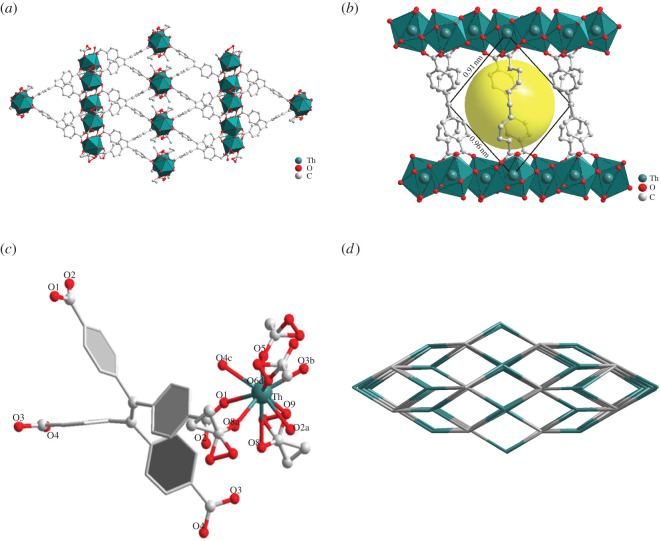
Schematic representation of compound **1** structure. (*a*) Three-dimensional porous structure. (*b*) Space-filling model. Parallelogram side lengths were calculated using the van der Waals radii. (*c*) Th(IV) coordination environment. (*d*) Topology framework with channels.

SCXRD revealed that compound **1** crystallizes in the monoclinic space group C2/c and contains nine-coordinated Th simultaneously bridged and chelated by H4TCPE (figure 1*a*). The whole complex has only one quadrilateral pore, 9.1 × 9.6 Å (diagonally) in size (see packing diagram in figure 1*b*). Th is coordinated by four carboxyl oxygens of four different ligands and five carboxyl oxygens from four CH_3_COO^−^ molecules (figure 1*c*). The bond length between Th and carboxylic O is 2.425(12)-2.508(14) Å, while between Th and acetic acid O it is 2.382(11)-2.737(9) Å. Its topology is flu, (4,8)-connected (figure 1*d*).

#### 3.1.2. Compound **2**

**Figure 2.**
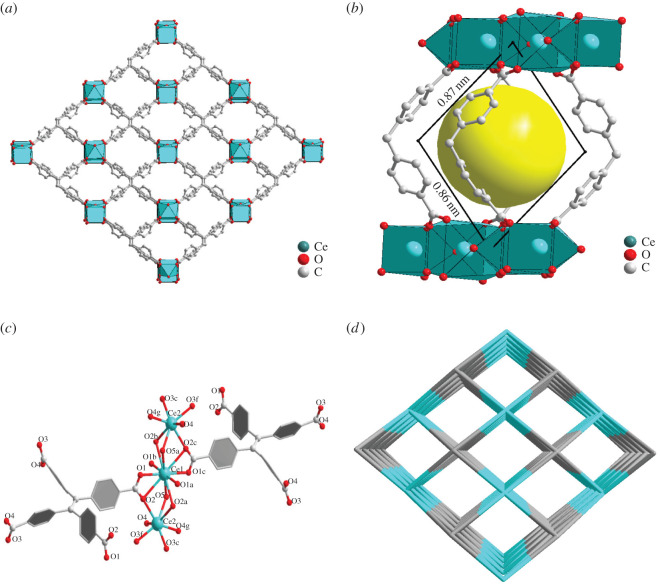
Schematic representation of compound **2** structure. (*a*) Three-dimensional porous structure. (*b*) Space-filling model. Parallelogram side lengths were calculated using the van der Waals radii. (*c*) Ce(IV) and Ce(III) coordination environments. (*d*) Topology framework with channels.

It was found that Ce(IV) located on both sides of the trinuclear cluster in compound **2** is reduced to Ce(III) by analysing the XPS data of Ce in compound **2** (see electronic supplementary material, figure S5). SCXRD revealed that compound **2** crystallizes in the monoclinic space group C2/m and contains 10-coordinate Ce (IV) and seven-coordinate Ce(III) units simultaneously bridged and chelated by H4TCPE (figure 2*a*). The whole complex has only one quadrilateral pore, 8.7 × 8.6 Å (diagonally) in size (see packing diagram in figure 2*b*). The three Ce atoms form a symmetrical trinuclear cluster Ce(IV)Ce(III)_2_(μ-OH)_2_(CO_2_)_4_(CO)_12_ with two Ce(IV)-O-Ce(III) bonds (figure 2*c*). The length of the bond between Ce and O atoms from the carboxyl groups is 2.370(9)–2.839(11), while the Ce(IV)-O-Ce(III) bond is equal to 2.514(13)-2.624(11) Å. The centre of the trinuclear cluster is sixfold node, while the ligand is 12-fold node (figure 2*d*).

### 3.2. Specific surface area and porosity

**Figure 3.**
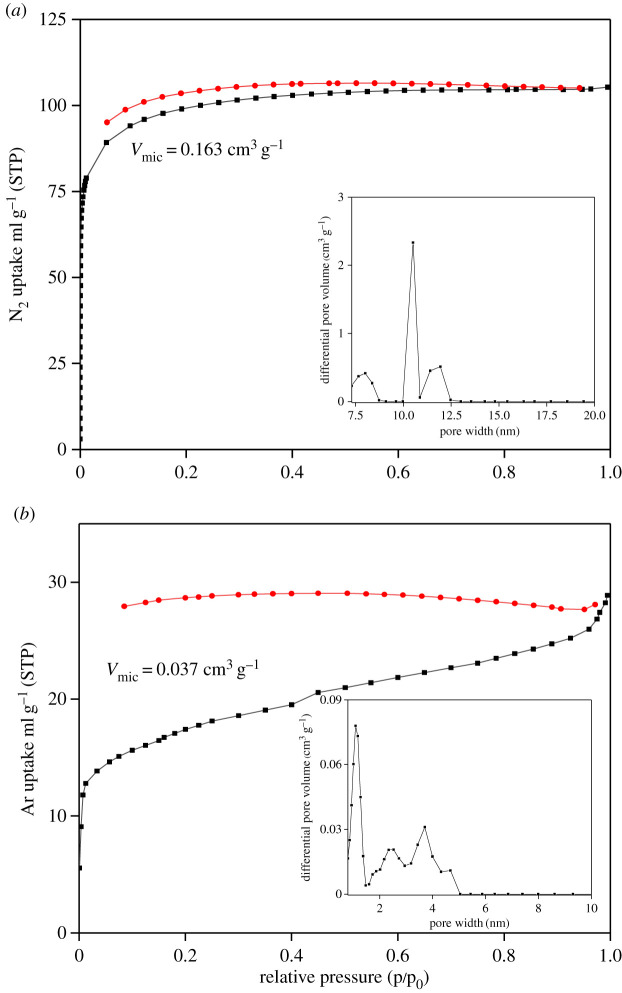
N_2_ (*a*) and Ar (*b*) adsorption/desorption isotherms and pore size distributions recorded for compound **1** (*a*) and **2** (*b*), respectively.

The surface area of compound **1** (equal to 308 m^2^ g^−1^) was similar to that of Th-TCPB-1, Th-TCPB-2 and Th-TCPB-3 compounds containing the same tetradentate ligand and possessing surface area in the 200–400 m^2^ g^−1^ range.^18^ The surface area of compound **2** was equal to 55 m^2^ g^−1^, which is much smaller than Ce-MOFs, such as Ce-CAU-24 [24]. The pores of compound **2** are so small that the less polar Ar was chosen. The structure was not destroyed by XRD and IR after activation (see electronic supplementary material, figures S1 and S2). We believe that the Ce(IV) trinuclear cluster affected the MOF pores (electronic supplementary material, figure S4). The micropore volumes of compounds **1** and **2** were equal to 0.163 and 0.037 cm^3^ g^−1^ (figure 3*a*,*b*), respectively. These values are consistent with the space occupied by solvent and crystalline water (contents of which were calculated from the TGA data described below performed by the NLDFT method) [25,26]. The pore sizes of compounds **1** and **2** were in the 7–13 Å and 0–5 Å ranges, respectively. Compound **2** has a small specific surface area and a large number of micropores, which results in the difference between its adsorption and desorption isotherms.

### 3.3. Thermal properties

**Figure 4.**
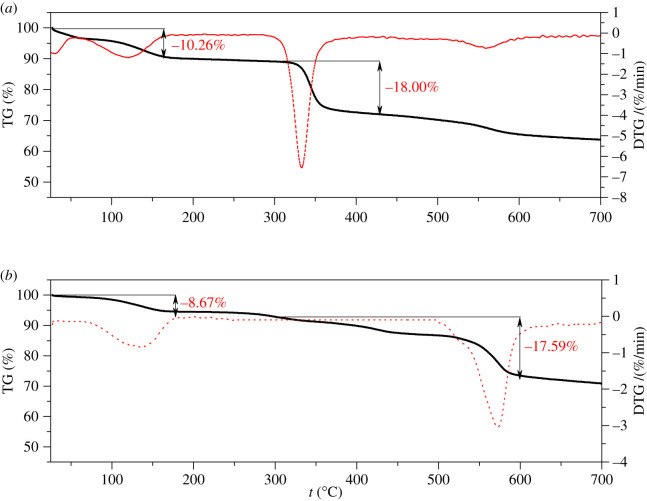
The TGA curves of compounds **1** (*a*) and **2** (*b*).

TGA curves of compounds **1** and **2** showed water and solvent molecule removal below 160 and 170°C, respectively (figure 4), with the corresponding weight losses equal to 10.26% and 8.67%. *In situ* XRD patterns (electronic supplementary material, figure S6) showed that the characteristic peaks of compounds **1** and **2** disappeared at 400°C and 600°C, respectively. Combined with TGA analysis, structural collapses of compounds **1** and **2** were observed at 343.8°C and 513.3°C. Compound **1** structure could not withstand temperatures as high as the previously synthesized Th-based MOFs [18], potentially because of the acetic acid molecule presence. At the same time, compound **2** demonstrated excellent structural thermal stability, especially relative to other Ce-based MOFs, which was attributed to the presence of the trinuclear Ce cluster [24].

### 3.4. Luminescence properties

The colours of compounds **1** and **2** under visible light are light blue and white. However, compound **1** emitted intense blue light upon exposure to the UV light (figure 4).

**Figure 5.**
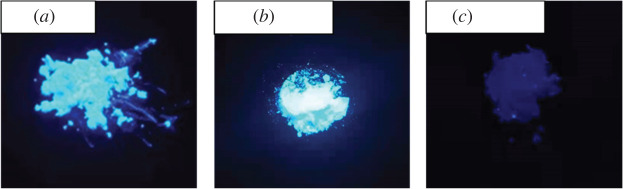
Photographs of powders of H4TCPE (*a*), compound **1** (*b*) and compound **2** (*c*) under exposure to a 365 nm UV light.

**Figure 6.**
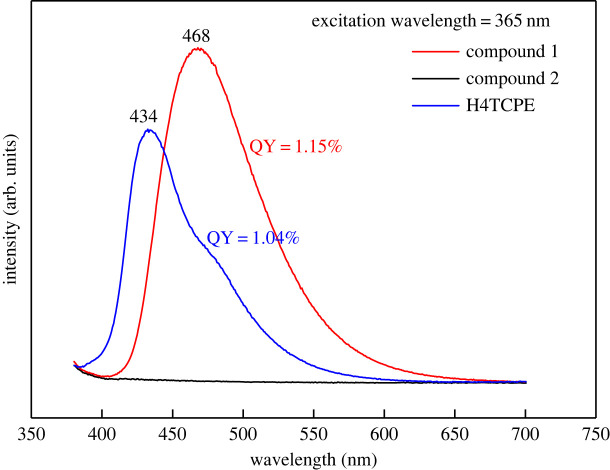
Fluorescence spectrum of compound **1** (red curve), compound **2** (black curve) and H4TCPE (blue curve) with the corresponding QYs.

Fluorescence spectra of compounds **1** and H4TCPE showed peaks with maxima at 468 and 434 nm, respectively, when exposed to the 365 nm UV light (figure 5). The QY of compound **1** (equal to 1.15%) was higher than of the H4TCPE ligand (which was equal to 1.04%). The fluorescence spectrum of compound **1** red-shifted relative to the corresponding spectrum of the H4TCPE ligand (figure 6). Compound **1** is an AIE-type chromophore whose organic ligand provides luminescence. Since Th is bonded to the H4TCPE ligand and four acetic acid molecules, the electron cloud density in the ligand is changed, which translates into this slight red-shift [27]. Thorium still has f-layer electrons after losing the four outermost electrons. Compared with cerium, the outermost electrons of thorium are farther away from the nucleus and require less energy for electronic transition. The fast rotation of the phenyl rings and partial twisting of the C=C bond quench are prevented by Th in the rigid matrix, resulting in enhanced luminescence properties [28–30].

There is enough space in the crystal structure of compound **2** for the free rotation of the benzene ring to cause self-quenching effect, so the luminescence is extinguished. Similar behaviour is observed for Ce-CAU-24 [23].

## 4. Conclusion

In this work, we synthesized two new MOFs by a simple procedure and under mild conditions. Compound **1**, which denoted Th_4_(TCPE)_2_(CH_3_COO)_8_, is a mono-nuclear MOF with space group C2/c. Compound **2**, denoted as Ce(IV)Ce(III)_2_(μ-OH)_2_(TCPE)_2_, is a trinuclear MOF cluster with space group C2/m, The atoms of Ce(IV) were partially reduced to Ce(III) in the reaction environment. The structure of compound **1** is stable up to 343.8°C. Its surface area and micropore volume are equal to 308 m^2^ g^−1^ and 0.163 cm^3^ g^−1^, respectively. Compound **2** is stable up to 513.3°C and possesses surface area and micropore volume equal to 55 m^2^ g^−1^ and 0.037 cm^3^ g^−1^, respectively. Compound **1** emits stronger pale green luminescence, originating from the ligand. Compound **2** did not exhibit any luminescing properties due to a self-quenching mechanism.

## Data Availability

Electronic supplementary material [31]: PXRD patterns, SEM micrographs, BET and pore size distribution data, XPS as well as CIF files. Accession Codes: CCDC (2 098 332–2 098 333) contains the supplementary crystallographic data for this paper.
